# Editorial: Molecular and physiological mechanisms driving phytoremediation

**DOI:** 10.3389/fpls.2025.1770267

**Published:** 2026-01-20

**Authors:** Azam Noori, Hamidreza Sharifan, Andrés Rodríguez-Seijo

**Affiliations:** 1 Department of Natural Sciences, Merrimack College, North Andover, MA, United States; 2 Department of Chemistry and Biochemistry, the University of Texas at El Paso (UTEP), El Paso, TX, United States; 3 Environmental Science and Engineering Program, University of Texas at El Paso, El Paso, TX, United States; 4 Sharifarm LLC, Research & Development, El Paso, TX, United States; 5 Departament of Plant Biology and Soil Science, Faculty of Sciences, Universidade de Vigo, Ourense, Spain; 6 Instituto de Agroecoloxía e Alimentación (IAA), Universidade de Vigo, Ourense, Spain

**Keywords:** emerging contaminant, heavy metal, PFAS, remediation, sustainable remediation

Phytoremediation is increasingly being repositioned from a low-input remediation option to a mechanistically informed, systems-based strategy for managing complex environmental contamination under contemporary stressors. Advances in plant molecular biology, rhizosphere ecology, and environmental chemistry have shifted the field toward a mechanistic understanding of how physiological regulation, gene networks, and plant–microbe interactions govern contaminant uptake, transformation, and stabilization across soil–water–plant continua. This renewed focus is driven by the growing prevalence of emerging pollutants, multi-contaminant mixtures, and climate-related stresses that challenge conventional remediation technologies (Kishk et al., 2025). Modern phytoremediation frameworks integrate mechanisms such as rhizodegradation, phytodegradation, phytoextraction, rhizofiltration, phytovolatilization, and rhizostabilization as interacting, mutually reinforcing functions within adaptive, nature-based remediation systems designed to deliver cost-effective and environmentally resilient outcomes (Sharma et al., 2023).

Interest in these approaches has increased in recent years because they are nature-based solutions that use plants to remediate contaminants in air, water and soils, and can be further enhanced by rhizosphere bacteria (Wang et al., 2022) or fungi (Khalid et al., 2021). Most of these species have strong potential both as biomass for energy generation and as resources for contaminant recovery through phytomining, a process increasingly highlighted in the context of a circular economy (Clemente et al., 2025; Ranđelović et al., 2025).

Despite advancements in phytotechnology, critical knowledge gaps continue to limit the predictive, scalable application of phytoremediation. Our understanding of how contaminants are transported, transformed, and sequestered at molecular, cellular, and whole-plant levels remains incomplete, particularly for emerging pollutants such as PFAS, nanomaterials, and antibiotic residues. Moreover, the interactive effects of multiple contaminants, plant genotype, and abiotic stressors, especially drought and elevated temperature, are poorly characterized, even though they fundamentally reshape contaminant bioavailability and plant physiological responses. The limited integration of omics-driven molecular insights with field-relevant physiological data also constrains our ability to design or engineer plants with enhanced remediation efficiency. Addressing these gaps is essential for moving from proof-of-concept experimentation toward reliable, climate-resilient phytoremediation strategies.

The contributions to this Research Topic address a range of technologies for reducing soil and water contamination through phytoremediation practices. Noori et al. evaluated the ability of duckweed (*Lemna minor*), a floating freshwater macrophyte, to remediate perfluorooctanoic acid (PFOA), one of the top five PFAS (Per- and polyfluoroalkyl substances) of concern, classified as a long-chain PFAS (C8) with an approximate half-life of 3.5 years in human blood. PFAS have gained increasing attention as emerging pollutants (Glüge et al., 2020) because of their widespread but incompletely characterized environmental distribution and their documented adverse effects on biota and humans, leading to their inclusion in multiple environmental guidelines. Noori et al. observed significant effects of PFOA exposure on duckweed, including toxicity symptoms such as reduced growth and chlorosis. Nonetheless, they also reported promising uptake of PFOA by duckweed, indicating its potential utility in aquatic phytoremediation.

Furthermore, two studies in this Research Topic examined aided metal phytoremediation using bacterial inoculants. Karna et al. assessed the role of *Bacillus cereus*, isolated from topsoil and rhizosphere of rabbitfoot grass (*Polypogon monspeliensis*), in enhancing selenium (Se) volatilization within soil-Indian mustard (*Brassica juncea*) systems, demonstrating the importance of plant-microbe interactions for effective Se phytoremediation. Building on similar principles, Kong et al. conducted pot experiments using soils from a copper mine to compare the effects of plant growth-promoting bacteria (PGPB) with non-PGPB inoculants on the indigenous rhizosphere and bulk-soil microbiomes of Indian mustard in heavy metal-contaminated soil. Their results showed that PGPB inoculants not only persisted in the rhizosphere but also actively integrated into microbial networks, strengthening associations with indigenous bacterial taxa.

Finally, Wang et al. investigated plant-specific B3 family genes in pearl millet (*Pennisetum glaucum*) under drought and high-temperature stress. Because these abiotic stresses can elicit physiological responses similar to those observed in plants exposed to high contaminants level, their findings may inform future applications of *P. glaucum* in both inorganic (Dias et al., 2024) and organic (Silva et al., 2023) phytoremediation.

Ranđelović et al. (2025) recently highlighted several key research directions for advancing metal phytoremediation, including: i) elucidating physiological and molecular mechanisms in higher plants; ii) developing and optimizing assisted phytoremediation using diverse techniques and approaches; iii) conducting long-term studies under authentic field conditions, or iv) valorizing biomass generated from phytoremediation.

Further research is needed to address emerging contaminants, such as antibiotics, plant protection products, and PFAS, in both aquatic and terrestrial environments. Equally important is the expansion of experimental work simulated global-warming conditions including drought and elevated temperatures. Such stressors can markedly influence contaminant adsorption-desorption dynamics, alter key soil processes, and modify plant performance, ultimately reshaping the effectiveness of phytoremediation strategies.

Collectively, the contributions to this Research Topic highlight the expanding toolbox of molecular, physiological, and biotechnological approaches available to advance phytoremediation. By integrating mechanistic studies, plant–microbe interactions, genetic engineering, and field-oriented investigations, this Research Topic underscores the transformative potential of modern plant science to address global contamination challenges. We hope this Research Topic will catalyze new collaborations, inspire innovative methodologies, and provide a foundation for developing resilient, nature-based solutions capable of meeting the demands of an increasingly complex environmental landscape.
